# Fecal Occult Blood Test and Gastrointestinal Parasitic Infection

**DOI:** 10.1155/2010/434801

**Published:** 2010-07-26

**Authors:** Majed H. Wakid

**Affiliations:** Department of Medical Laboratory Technology, Faculty of Applied Medical Sciences, King AbdulAziz University-Jeddah, P.O. Box 80324, Jeddah 21589, Saudi Arabia

## Abstract

Stool specimens of 1238 workers in western region of Saudi Arabia were examined for infection with intestinal parasites and for fecal occult blood (FOB) to investigate the possibility that enteroparasites correlate to occult intestinal bleeding. Direct smears and formal ether techniques were used for detection of diagnostic stages of intestinal parasites. A commercially available guaiac test was used to detect fecal occult blood. 47.01% of the workers were infected with intestinal parasites including eight helminthes species and eight protozoan species. The results provided no significant evidence (*P*-value = 0.143) that intestinal parasitic infection is in association with positive guaiac FOB test.

## 1. Introduction

Human infections due to intestinal parasites caused by helminths and protozoa are among the most prevalent infections in developing and tropical countries causing a significant morbidity and mortality [1]. Human intestinal helminths include the nematodes (roundworms), the trematodes (flukes), and the cestodes (tapeworms). The human intestinal protozoa include nonpathogenic and pathogenic amoebae, nonpathogenic and pathogenic flagellates, and a pathogenic ciliate, in addition to human intestinal coccidian parasites.

Fecal occult blood (FOB) refers to a nonvisible blood in the stool. Although the FOB test was developed to specifically screen for colon cancer [2, 3], there are various causes of positive FOB including infection with some intestinal parasites [4, 5]. From parasitologic point view, most of the published studies concerned with determination of FOB in patients infected with *Trichuris trichiura*, Hook worm, *Schistosoma* spp. and *Entamoeba histolytica* [6–14]. The aim of the present study was to determine if there is correlation between positive FOB and any of the common intestinal parasitic infection in Saudi Arabia. 

## 2. Materials and Methods

### 2.1. Samples Collection

Stool samples were obtained randomly from 1238 workers in the western region of Saudi Arabia. Each worker was provided with a clean stool container and instructions for collection.

### 2.2. Parasitologic Examination

The direct smears and formal ether concentration techniques were used for detection of diagnostic stages of enteric parasites [15, 16].

*Direct stool smears* were performed by emulsifying 2 mg of stool uniformly in a drop of saline or iodine on a microscope slide, then covered with cover glasses and scanned microscopically.

*Formal ether concentration technique* was performed by emulsifying 2 g of stool in 15 mL of 10% (v/v) formal-saline. In unpreserved specimens, the suspension was allowed to stand for 30 min, then strained through two layers of gauze into a 15 mL conical centrifuge tube and centrifuged at 2000 rpm for 5 min. When needed, washing step was repeated until supernatant becomes clear. The sediment was resuspended with 10 mL of 10% (v/v) formal-saline, then 3 mL of diethyl ether was added. The tube was shaken vigorously for 30 sec and centrifuged at 2000 rpm for 5 min. The fecal debris layer was loosened by wooden applicator stick and the tube rapidly inverted to discard the top three layers while the sediment remained at the bottom. A drop of iodine was mixed with the sediment, then transferred to a microscope slide, covered with a cover glass, and scanned microscopically.

### 2.3. Occult Blood Test

Each stool sample was processed to detect occult blood using a guaiac-based test (Hema-Screen, Stanbio, Texas, USA) by spreading thin smear of stool according to the manufacturer instructions. Hema-Screen can detect 10 mg of hemoglobin per gram of feces. A positive reaction is indicated by the appearance of a blue-green color between 30 seconds and two minutes after addition of two drops of developer reagent. The occult blood tests were achieved by a technologist blinded to parasitologic results.

### 2.4. Statistical Analysis

Data analysis was performed using SPSS V17.0 statistic Package. The Chi square test was used for determining the significance of association between intestinal parasitic infection and FOB test finding. A *P* *-*value level of significance was 0.05.

The study was approved by the Research and Ethics Committee of the Faculty of Applied Medical Sciences, King Abdulaziz University, Jeddah, Saudi Arabia.

## 3. Results

The enrolled 1238 workers in this study were males within the age of 17–70. Six hundred and fifty six (52.99%) cases without intestinal parasitic infection were the control of the study.

Five hundred and eighty two (47.01%) were infected with intestinal parasites. These parasites included hookworms, *Trichuris trichiura*, *Ascaris lumbricoides*, *Hymenolepis nana, Schistosoma mansoni, Strongyloides stercoralis, Enterobius vermicularis, Taenia saginata, E. histolytica, Giardia lamblia, Blastocystis hominis,* and several nonpathogenic intestinal protozoan parasites (*Entamoeba coli, Endolimax nana, Entamoeba hartmanii, Iodamoeba butschlii,* and *Chilomastix mesnili*).

Of 582 infected cases, the positive rate for occult blood was 130 (22.43%). There were 170 (25.91%) positive fecal blood results in the 656 control group (Table 1). 

There was no significant association between positive FOB test and infection with intestinal parasites (*P* = 0.0143). Results of FOB for each detected parasitic infection alone or in presence of other parasites as mixed infection are illustrated in Table 2.

## 4. Discussion

Detecting fecal occult blood by a guaiac-based test is still the most widely used test in medical laboratories in Jeddah. This test depends on the peroxidase-like activity of hemoglobin in catalyzing the oxidation by peroxide of a chromogen. Dietary and medication restrictions are recommended with this test to decrease false positive and false negative results [5]. Immunochemical FOB test, which does not require dietary restrictions, is more expensive and not used widely in Jeddah. An earlier study showed that both tests had a similar positive predictive value [17]. According to that, guaiac-based test was suitable for our design during this study to resemble the situations during routine analysis in medical laboratories in Jeddah, regardless of dietary and medication restrictions, which is not followed by the majority of ordinary population in Jeddah and regardless of infection intensity, which is not performed with routine FOB testing.

During this study several pathogenic and nonpathogenic enteroparasites were detected and investigated for correlation to FOB positivity. There was no significant difference in FOB positivity between infected and noninfected samples (Tables 1 and 2).

The common parasites which were detected and can cause dysentery or blood loss were *Trichuris trichiura,* hookworms, *Schistosoma mansoni,* and* E. histolytica*. Previous studies illustrated that occult blood is not correlated with *T. trichiura* infection, unless there is a *Trichuris* dysentery syndrome (TDS) [6, 7, 10]. Similarly for hookworms, they do not lead to a significant occult gastrointestinal bleeding unless high-burden infection occurs [11–13]. Same observation was reported concerning *Schistosoma* spp. that infects humans and causes anemia [9, 14, 18–20]. A previous study reported four asymptomatic cases with positive FOB test and amebic colitis due to *E. histolytica* [8].

In the present study, all samples with* E. vermicularis* and* T. saginata* reacted negatively with FOB test. In a previous study,* E. vermicularis* (pinworm) gave positive FOB but in a case with colon carcinoma [21].

The importance of the recognition of nonpathogenic (commensals) amoebae and flagellates lies in the fact that these organisms are indicative of fecal-oral transmission having occurred. When these organisms are found in stool samples, it is important to be on the lookout for the possible presence of pathogenic species. *B. hominis* represented the highest infection rate (19.79%); however, describing this parasite as pathogenic is still controversial [22–24].

In summary, the present study demonstrated that positive guaiac-based FOB test during routine analysis in Jeddah could not be correlated with the intestinal parasitic infection. However, as guaiac-based FOB test is widely used in Jeddah, more awareness for the public about importance of restricting some foods and drugs intake prior FOB test is needed.

## Figures and Tables

**Table 1:** FOB finding in negative (control) and positive parasitic infections.

	Parasitic infection		
	Negative (*n* = 656)	Positive (*n* = 582)	
+Ve FOB	170 (25.91%)	130 (22.43%)	*P*-value = 0.0143
−Ve FOB	486 (74.09%)	452 (77.57%)	

**Table 2:** FOB results in correlation with infection of detected parasites. *n* = number of infected cases. *All samples were negative for FOB test.

Parasite (*n*)	(Single infection)		(Mixed infection)		*P*-value
	+Ve FOB	−Ve FOB	+Ve FOB	−Ve FOB	
Hookworm (184)	32	67	35	50	0.213
*T. trichiura* (123)	14	37	19	53	0.896
*A. lumbricoides* (11)	2	4	1	4	0.621
*H. nana* (10)	0	4	0	6	*
*S. mansoni* (14)	2	1	4	7	0.347
*S. stercoralis* (20)	1	4	3	12	1.0
*E. vermicularis* (2)	0	0	0	2	*
*T. saginata* (2)	0	1	0	1	*
*E. histolytica* (43)	1	0	14	28	0.167
*G. lamblia* (39)	4	17	3	15	0.847
*B. hominis* (245)	16	109	17	103	0.754
Nonpathogenic protozoa (156)	12	33	19	92	0.176
